# MicroRNAs in systemic lupus erythematosus: Molecular networks, pathway dysregulation, and therapeutic targeting strategies

**DOI:** 10.1016/j.biotno.2026.07.002

**Published:** 2026-09-01

**Authors:** Mohamed M. Sadaty, Rana Mohamed, Emad El-Zayat, Salma M. Mekhemer, Amira El-Ansary, Nahla O. Mousa

**Affiliations:** a Department of Medical Laboratory Technology, Faculty of Applied Health Science Technology, Misr University for Science and Technology, Giza, 3237101, Egypt; b Department of Biotechnology, Faculty of Science, Cairo University, Giza, 12613, Egypt; c Department of Internal Medicine, Faculty of Medicine, Misr University for Science and Technology, Giza, 3237101, Egypt

**Keywords:** Systemic lupus erythematosus (SLE), MicroRNAs (miRNAs), Immune dysregulation, Therapeutic

## Abstract

Systemic lupus erythematosus (SLE) is a complex autoimmune disorder characterized by immune dysregulation, chronic inflammation, and multi-organ involvement. Its pathogenesis is multifactorial, involving genetic, epigenetic, and environmental factors that collectively contribute to the loss of immune tolerance. In recent years, considerable attention has focused on epigenetic mechanisms, particularly microRNAs (miRNAs), which function as post-transcriptional regulators of gene expression and play pivotal roles in immune cell differentiation and function. Emerging evidence suggests that dysregulated expressions of specific miRNAs, such as miR-146a and miR-155, may contribute to aberrant immune responses, including altered T- and B-cell activity and cytokine production in SLE. These alterations have been associated with disease activity and immunopathological processes. In addition, circulating miRNAs have been investigated as potential non-invasive biomarkers for diagnosis and disease monitoring. Advances in high-throughput technologies have facilitated the identification of miRNA expression profiles associated with SLE; however, variability between studies and limited clinical validation remain challenges. While miRNA-based therapeutic strategies are being explored, their clinical application is still under investigation.

This review highlights the role of miRNAs in SLE pathogenesis, their potential as biomarkers, and their emerging relevance as therapeutic targets, while also addressing current limitations and challenges in clinical translation.

## 1 Introduction

SLE is a chronic autoimmune illness that can affect multiple organs in the body. SLE develops when the immune system, which normally protects the body against infections and other diseases, mistakenly attacks its own tissues. This attack causes inflammation and, in some cases, lasting tissue damage, which can be widespread affecting the skin, joints, heart, lungs, kidneys, circulating blood cells, and brain. ^1^

Beyond its systemic manifestations, SLE involves inflammation of connective tissues, including cartilage and vascular endothelium, which provide strength and flexibility to structures throughout the body. The disease manifests with a broad spectrum of clinical manifestations, including renal, cardiac, hematologic, integumentary, gastrointestinal, and even mental disorders. SLE is identified by the malar (butterfly) rash, which is defined as an erythematous, hyperpigmented, maculopapular rash over the cheekbones and nasal bridge while typically sparing the nasolabial folds. ^2^

The complex immunopathogenesis of SLE is driven by dysregulation of multiple interconnected signaling pathways, including type I interferon responses, Toll-like receptor (TLR) activation, and aberrant T-cell and B-cell signaling. Emerging evidence indicates that miRNAs play a pivotal role in modulating these pathways at the post-transcriptional level. ^3^

miRNAs can simultaneously regulate multiple target genes within key immune networks, thereby acting as critical regulators of immune homeostasis. In SLE, altered expression of specific miRNAs has been associated with enhanced inflammatory signaling, defective immune tolerance, and sustained autoantibody production. Given their ability to fine-tune complex immune responses, miRNAs represent a mechanistic link between epigenetic regulation and immune dysfunction in SLE. This highlights their potential not only as biomarkers but also as promising targets for therapeutic intervention. ^4^

## 2 Methodology

This review was conducted using a systematic literature search strategy to ensure comprehensive identification and coverage of relevant studies. Electronic databases including PubMed, Scopus, and Web of Science were searched for articles published up to 2026.

The search terms included combinations of the following keywords: systemic lupus erythematosus, SLE, microRNA, miRNA, epigenetics, biomarkers, and therapeutic targeting.

Inclusion criteria comprised original research articles, clinical studies, and relevant review articles focusing on the role of miRNAs in SLE pathogenesis, diagnosis, or therapy. Studies not published in English or lacking sufficient methodological clarity were excluded.

The selected studies were screened based on relevance, study design, and contribution to the topic. Evidence was qualitatively synthesized, and miRNAs were categorized according to their functional roles and strength of supporting evidence.

Gene sets were curated from published studies reporting experimentally validated miRNA–target interactions in SLE. Genes consistently reported across multiple studies were prioritized. Protein–protein interaction (PPI) networks were constructed using the STRING database to explore functional relationships among the identified genes. Functional enrichment analysis was performed using Gene Ontology (GO) and KEGG pathway databases to identify significantly enriched biological processes and pathways.

miRNA–target interactions were collected from publicly available databases, including miRTarBase for experimentally validated interactions and TargetScan for predicted targets.

## 3 Overview of SLE

### 3.1 Etiology of SLE

The etiological mechanisms underlying SLE remain incompletely understood. The combination of multiple susceptibility elements, such as environmental, hormonal, and genetic factors, makes it more heterogeneous and complex. Genetic and epigenetic alterations have been recognized as governing the immunobiology of lupus by environmental modifications such as diet and nutrition. ^5^

### 3.2 Immunopathogenesis of SLE

At the immunological level, SLE represents a prototype of systemic autoimmunity, characterized by persistent immune dysregulation and chronic inflammation. SLE immunopathogenesis is complex, with several interrelated processes that contribute to illness genesis and progression. The development of autoantibodies against self-nuclear antigens is a defining characteristic of SLE. These autoantibodies produce immune systems that deposit in different tissues, causing inflammation and tissue damage. ^6^

**Fig. 1:**
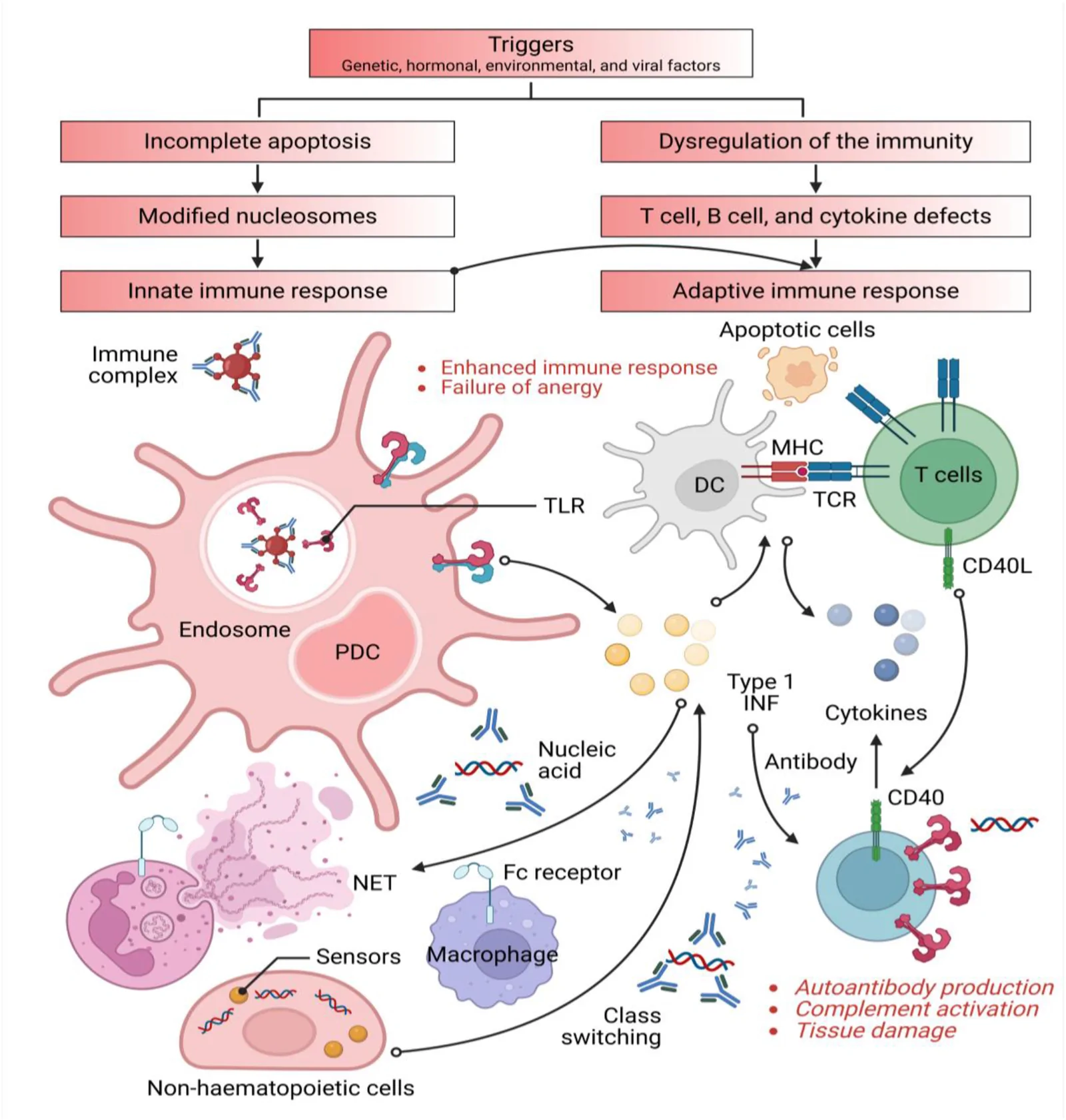
Systemic lupus erythematosus pathogenesis.

The pathogenesis of SLE is regulated by a cascade of immunological dysregulation that includes both innate and adaptive components. The disease process begins when genetic, epigenetic, hormonal, and environmental variables alter immunological homeostasis, activating Toll-like receptors (TLRs) in the innate immune system. This activation increases the production of type I interferons (IFNs), which are key cytokines in enhancing the autoimmune response, ^7^ **a**s shown in Fig. 1.

It is driven by mechanisms that excessively activate the immune system. Predisposing factors—including genetic, epigenetic, hormonal, and environmental influences—can either act as, or stimulate the production of immunoreactants that initiate Toll-like receptor (TLR) signaling within the innate immune system, leading to increased synthesis of type I interferons (IFNs). These factors may also modulate T-cell and B-cell development and maturation within the adaptive immune system, resulting in excessive autoantibody production. Type I IFNs serve as a central hub linking innate and adaptive immunity, being produced predominantly by innate lymphoid cells (ILCs). In turn, type I IFNs directly promote B-cell activation, driving the overproduction of autoantibodies that contribute to SLE pathology. Additionally, ILCs can present antigens to T-cells through major histocompatibility complex (MHC) molecules, thereby activating T-cells that express CD40 ligand (CD40L). This CD40L–CD40 interaction with B-cells further amplifies autoantibody production, exacerbating disease progression.

#### 3.2.1 STRING-based protein–protein interaction network highlights core immune pathways in SLE

**Fig. 2:**
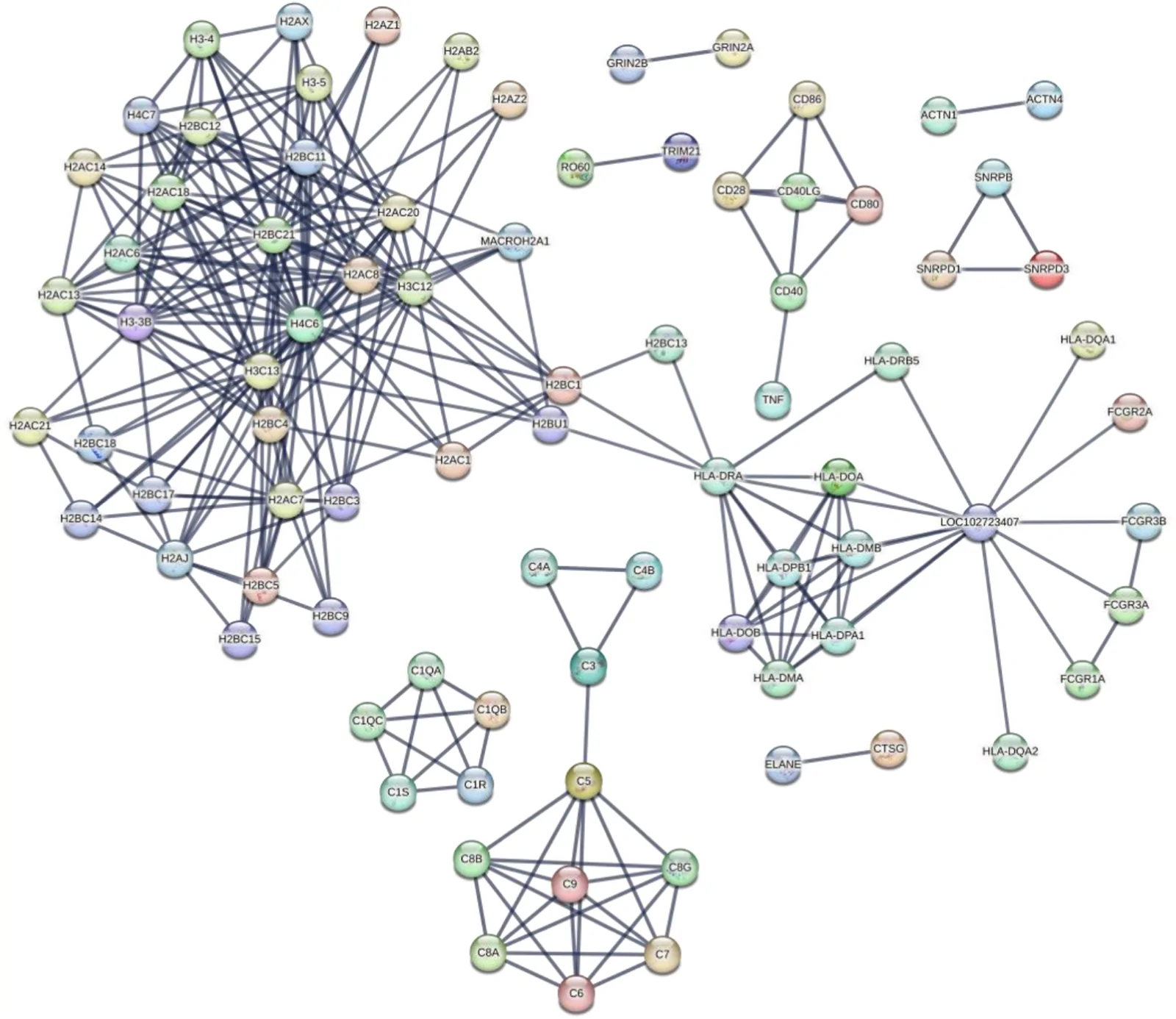
STRING protein–protein interaction (PPI) network of SLE-related proteins.

To supplement the mechanistic overview of SLE immunopathogenesis, a protein-protein interaction (PPI) network was created using the STRING database at the highest confidence level. The resulting network shows substantial interconnectedness among immune-related proteins, highlighting the multi-pathway character of SLE. Prominent clusters correspond to antigen processing and presentation, complement activation, Fc receptor-mediated immune complex handling, co-stimulatory signaling, and nuclear autoantigens, emphasizing SLE's systemic immunological dysregulation. (STRING database), as shown in Fig. 2.

The interaction network was constructed using the STRING database (*Homo sapiens*) with the highest confidence score (interaction score ≥0.900). Nodes represent proteins and edges indicate high-confidence functional associations supported by multiple evidence channels. The network reveals interconnected immune modules involving MHC class II antigen presentation, complement cascade, Fc receptor signaling, co-stimulatory pathways, inflammatory mediators, and nuclear autoantigens, highlighting the complex immune architecture underlying SLE pathogenesis. This figure was generated by the STRING database at the highest confidence level (0.900).

### 3.3 Risk factors for SLE

**Fig. 3:**
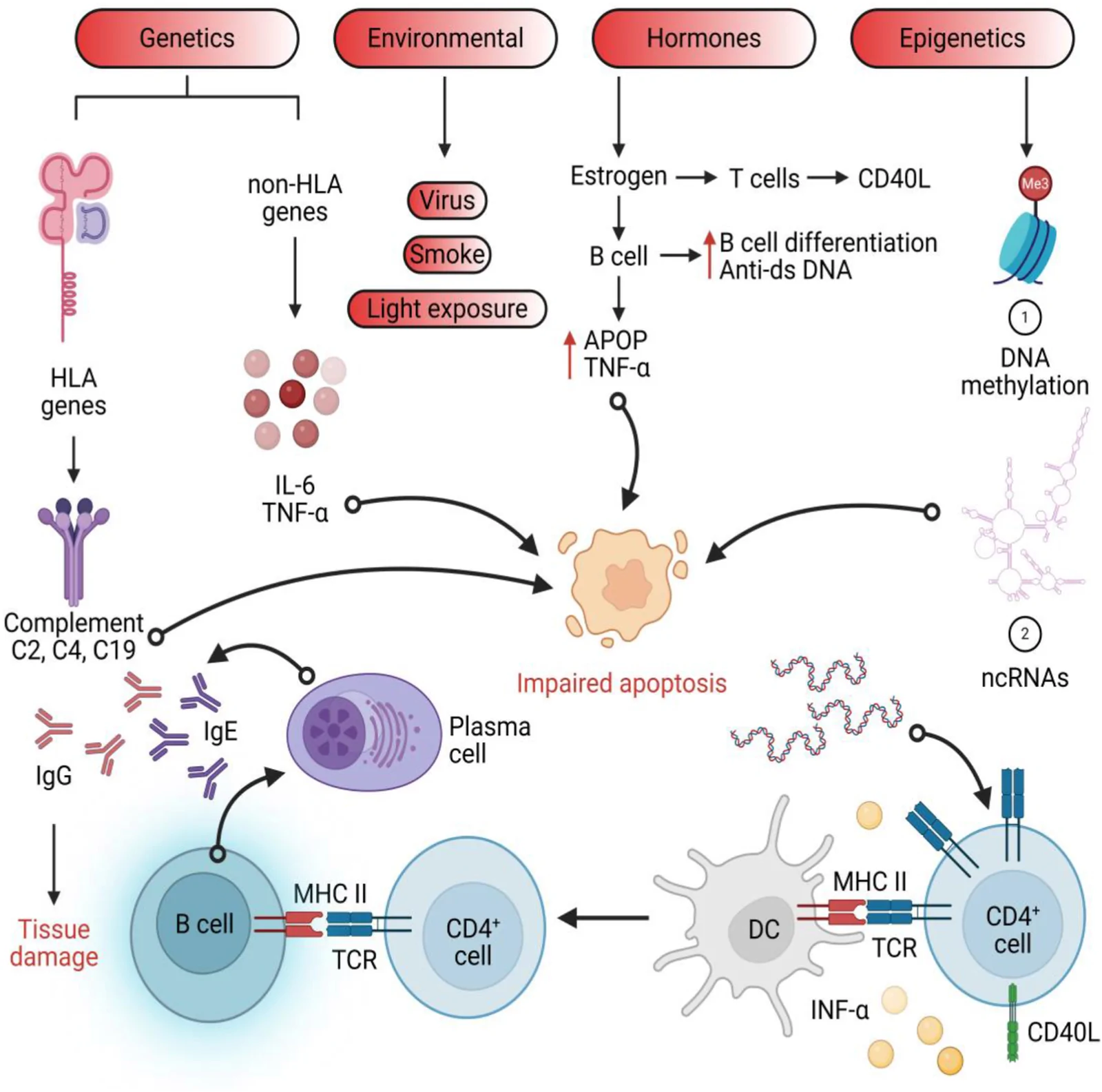
Risk factors affect SLE: genetics, environment, hormones, and epigenetics.

Several risk factors comprising genetic as well as non-genetic elements provide a hostile environment for the change towards autoimmunity, **a**s shown in Fig. 3.

#### 3.3.1 Genetic factors

Numerous genetic studies in different SLE populations have found several susceptibility loci. SLE has a wide range of clinical and laboratory symptoms. As proven, certain laboratory characteristics are related to disease phenotypes of varying severity. Similarly, in recent years, a relationship between phenotypes and genetic variants has been established, giving the prospect of elucidating distinct processes and pathways responsible for illness presentations. ^8^

#### 3.3.2 Environmental factors

Environmental risk factors play a crucial role in the development and progression of SLE. Smoking is strongly associated with an increased risk of SLE, particularly among current smokers and individuals positive for SLE-specific autoantibodies, although the risk declines following prolonged smoking cessation. Reproductive and hormonal factors also contribute to disease susceptibility, as SLE predominantly affects women of reproductive age and is associated with conditions such as polycystic ovarian syndrome, premature ovarian failure, preeclampsia, and adverse pregnancy outcomes. Dietary factors influence disease activity, with balanced diets rich in vitamin D, antioxidants, omega-3 fatty acids, and high-quality proteins being associated with improved immune regulation and reduced disease severity. Additionally, infections are considered important environmental triggers, with pathogens such as Epstein–Barr virus, cytomegalovirus, human endogenous retroviruses, and certain bacterial species implicated in initiating autoimmune responses in genetically susceptible individuals. 9, 10, 11, 12

### 3.4 Clinical picture of SLE

**Fig. 4:**
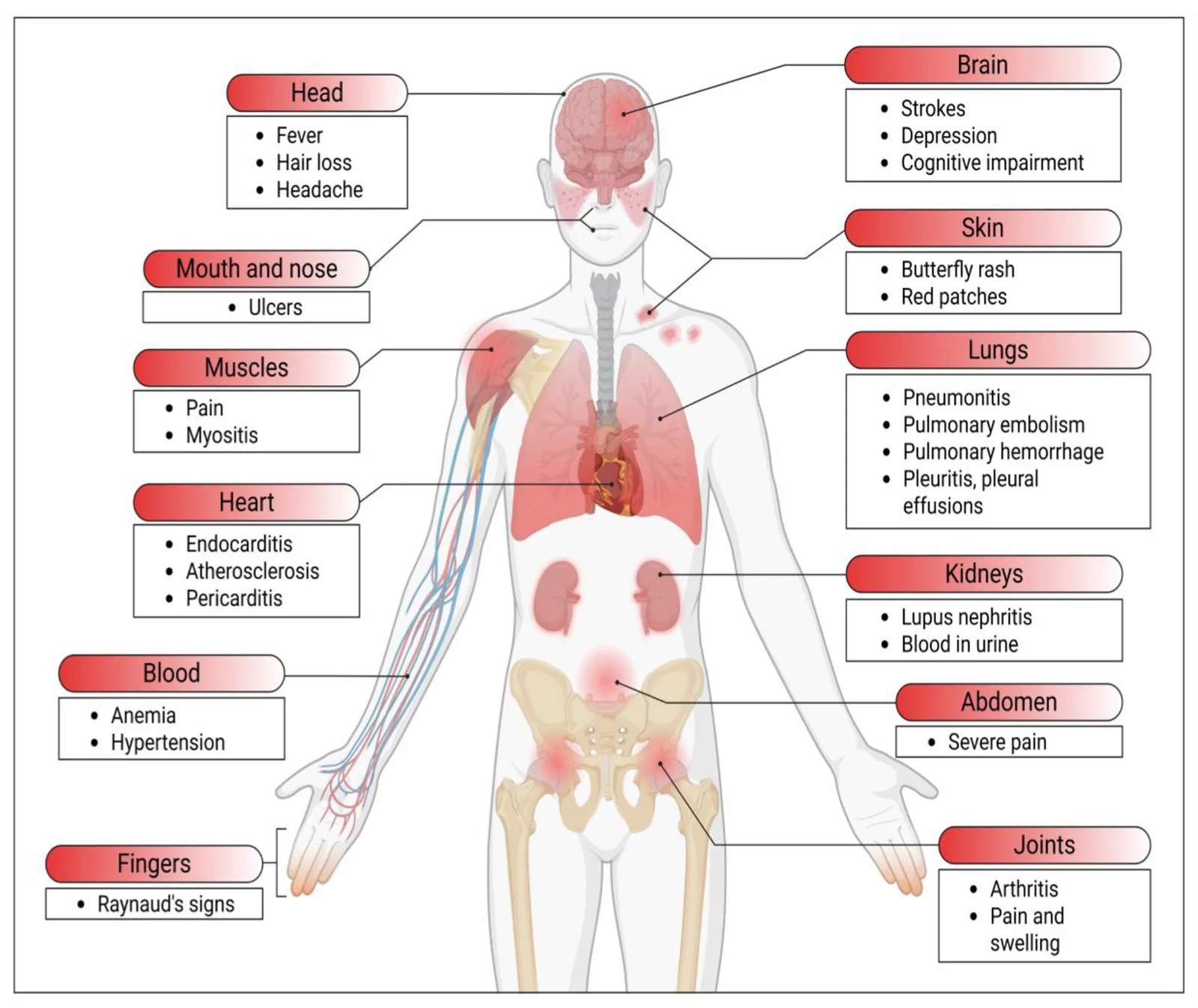
Summary of the clinical manifestations of SLE.

There is a difference between the clinical presentation of SLE with an early and late onset. While late-onset SLE is characterized by arthritis, leukopenia, and thrombotic complications, it is less usual to see skin symptoms, photosensitivity, nephropathy, vasculitis, and involvement of the central nervous system as shown in Fig. 4. It takes a long time for late-onset SLE to be diagnosed, and the existence of other diseases dictates the course of treatment. ^13^

## 4 Epigenetics and SLE

Epigenetics is a novel area of research in SLE. The term ‘epigenetics’ was originally associated with heritable changes in gene activity that occur without alterations of the genetic code. This term is more often used to describe chromatin related regulatory mechanisms that do not involve changes in the nucleotide sequence, regardless of whether such imprinting is strictly heritable. ^14^

The effect of epigenetics in SLE ranges from contributing to complex disease mechanisms to identifying biomarkers for early diagnosis and response to therapy. Epigenetic mechanisms may serve as a dynamic link between environment, genotype and phenotype. Accumulating evidence has demonstrated that aberrant epigenetic modification plays a pivotal role in triggering the dysregulation of T-cell activation, leading to the incidence of SLE. ^15^

Within the epigenetic factors participating in SLE, miRNA playing an important role in many biological processes, are potential therapeutic targets for many autoimmune/inflammatory diseases, including SLE. ^16^

Altered miRNA expression has been associated with enhanced secretion of proinflammatory cytokines and activation of multiple inflammatory signaling pathways, as well as other processes that maintain the vicious cycle of autoimmunity. Moreover, miRNA also shows considerable potential for not only diagnostic (circulating miRNA in serum are attractive biomarkers) but also therapeutic applications. ^17^

## 5 miRNA as biomarkers

miRNA may have great potential for use as clinical biomarkers because easy, noninvasive detection in various medical conditions is possible. Circulating miRNAs have been shown as biomarkers in various diseases where they also are of prognostic relevance. There is now growing evidence that circulating miRNA also can be used as biomarkers in SLE. miRNAs were evaluated as potential markers to distinguish people with or without SLE. ^18^

The remarkable stability of miRNA in bodily fluids, such as serum, plasma, and urine, makes them outstanding candidates for diagnostic and prognostic purposes. Unlike typical protein biomarkers, miRNAs can reflect unique molecular and immunological processes, providing information about disease pathophysiology. Several studies have found that different miRNA expression profiles are linked with SLE onset and activity. For example, miR-21, miR-146a, and miR-155 are frequently increased in individuals with active illness and have been linked to abnormal T-cell and B-cell signaling. In contrast, miR-125a and miR-31 are frequently downregulated, which correlates with poor immunological tolerance. Elevated miR-146a levels have been demonstrated to regulate the type I interferon pathway, which is characteristic of SLE immunopathogenesis. ^19^

## 6 Role of miRNAs in the immune system and autoimmune diseases

### 6.1 miRNA and immune system

SLE is a well-known autoimmune disease, and both innate and adaptive immunities are the crucial steps for SLE development. Alternations of miRNA level can control the differentiation and immunological functions of various immune cells (monocytes, macrophages, and T-cells). ^8^

Numerous alterations in miRNA expression in these cells in SLE patients have been reported. Chronically activated T-cells are the trigger and key to SLE. They are also the crucial link in inducing and aggravating SLE immunological inflammatory response. They can induce the activation of macrophages. A great number of studies have confirmed that there are various miRNA expressions modulating T-cells. ^20^

### 6.2 miRNA and autoimmune diseases

Autoimmune diseases involve a complex dysregulation of immunity, and they reflect the interplay between environment and genetic factors. Autoimmune diseases include many members [e.g., rheumatoid arthritis (RA) and SLE], and most of them are classified according to what organs and tissues are targeted by the damaging immune response.^21^ Serum miRNA have served as potential biomarkers for detecting various diseases. ^22^ miRNA expression patterns are different in inflamed and non-inflamed ideal mucosa of patients with Crohn's disease (CD), and dysregulated miRNA may be responsible for CD pathogenesis. ^23^

## 7 Overview of miRNA dysregulation

Several miRNAs have been reported to exhibit differential expressions in SLE. For example, miR-21 and miR-155 are frequently upregulated in SLE patients and are associated with increased inflammatory signaling and disease activity. In contrast, miR-146a has been reported to be downregulated in certain immune cell subsets, contributing to dysregulation of the type I interferon pathway. However, variability between studies exists, as some reports have shown differential expression patterns depending on sample type and disease stage, ^8^ as shown in Table 1.

**Table 1:** Differential expression of miRNAs in SLE.

Evidence	Function	Expression in SLE	miRNA
Clinical ^24^	promotes inflammation	↑ Upregulated	**miR-21**
Clinical ^25^	immune activation	↑ Upregulated	**miR-155**
Experimental ^26^	regulates IFN pathway	↓ Downregulated (in T-cells)	**miR-146a**

## 8 miRNA regulation of key molecular pathways in SLE

miRNAs are key regulators of multiple molecular pathways implicated in SLE pathogenesis. One of the most prominent pathways is the type I interferon (IFN) signaling cascade, where dysregulated miRNAs such as miR-146a and miR-155 have been shown to modulate inflammatory responses. ^27^

In addition, miRNAs play critical roles in Toll-like receptor (TLR) signaling, influencing downstream activation of NF-κB and the production of pro-inflammatory cytokines. Dysregulation of these pathways contributes to the chronic inflammatory state observed in SLE. ^28^ Furthermore, miRNAs regulate both T-cell and B-cell function, affecting immune tolerance, autoantibody production, and lymphocyte activation. These findings highlight the central role of miRNAs as modulators of interconnected signaling networks in SLE. ^29^

## 9 Network and pathway-based analytical approach

To provide a systems-level perspective on the molecular mechanisms underlying systemic lupus erythematosus, an integrative network-based analytical approach was applied. Genes previously reported to be associated with SLE and immune dysregulation were compiled from the literature and used as input for protein–protein interaction (PPI) analysis. High-confidence interaction networks were generated using the STRING database (*Homo sapiens*), applying the highest confidence threshold (interaction score ≥0.900) to minimize spurious associations. ^30^

To integrate epigenetic regulation with immune network architecture, curated and predicted miRNA–target interactions were mapped onto the identified hub genes. Chord diagrams were generated using RAW Graphs to visualize the convergence of circulating miRNAs on central immune pathways. This analytical strategy was employed as an integrative, in-silico approach to contextualize previously reported molecular data rather than to infer novel experimental causality. ^31^

## 10 miRNAs as potential biomarkers in SLE

**Fig. 5:**
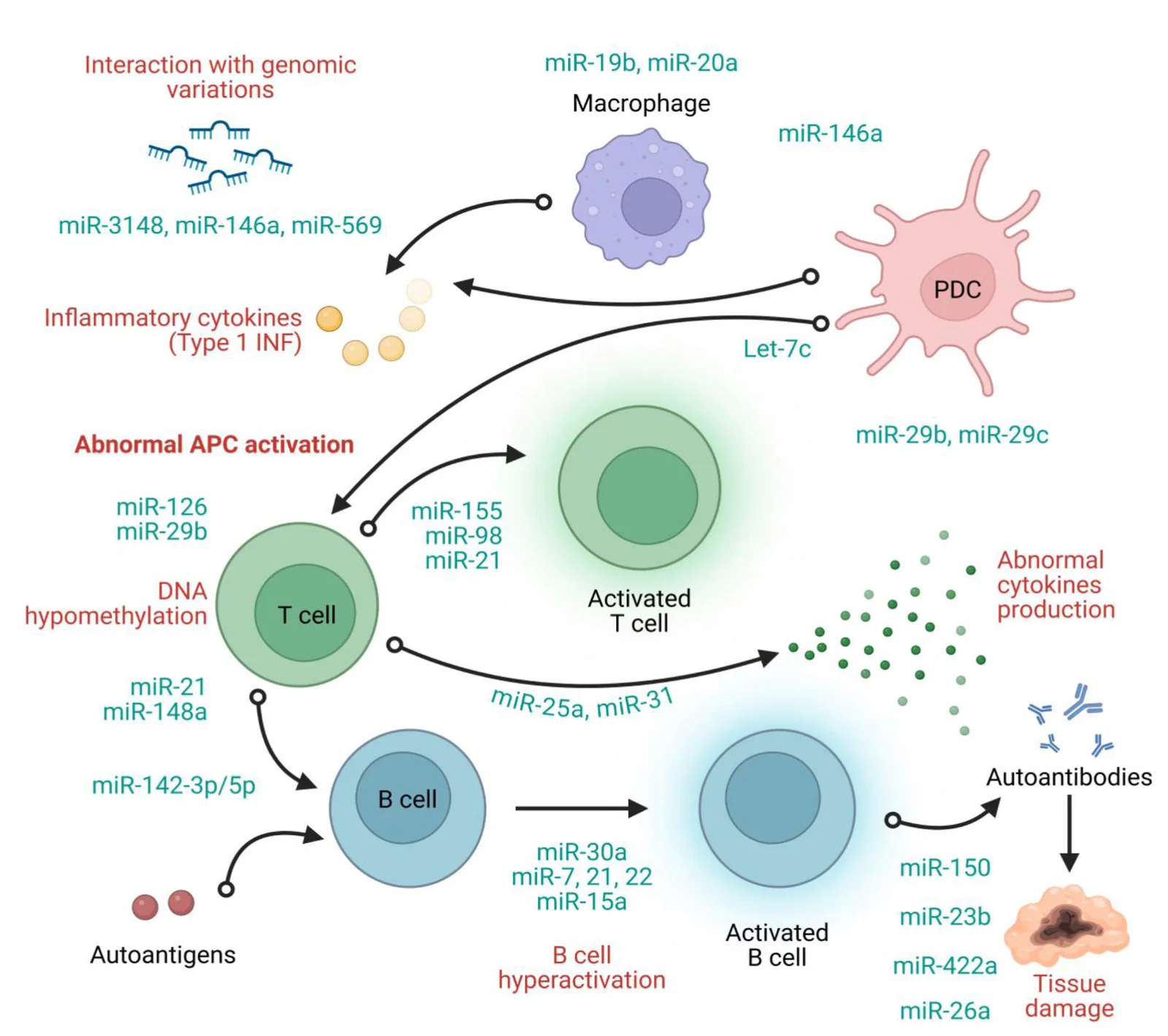
Functional role of miRNAs in SLE.

A series of miRNAs were found to be uncontrolled in subsets of SLE patients, leading to the production of proinflammatory cytokines and the activation of leukocytes as shown in Fig. 5 & Table 2 Numerous miRNAs related to inflammatory cytokines have been identified to date, and attempts have been made to use them in SLE treatment. ^32^

**Table 2:** miRNAs Implicated in Systemic Lupus Erythematosus (SLE) Pathogenesis.

Strength of evidence	Evidence Source	Interaction Type	Mechanistic contribution to SLE	Representative target genes	miRNA
Strong	miRTarBase	Experimentally validated	It functions as a negative feedback regulator of Toll-like receptor and type I interferon signaling; lower expression promotes pro-inflammatory cytokine release and immunological activation. ^33^	TRAF6, IRAK1	**miR-146a**
Strong	miRTarBase	Experimentally validated	Promotes T- and B-cell activation, differentiation, and cytokine release; overexpression causes immunological hyperreactivity and tissue damage. ^34^	SOCS1, SHIP1, PU.1	**miR-155**
Strong	miRTarBase	Experimentally validated	Supports lymphocyte survival and Th17 polarization; overexpression promotes autoantibody production and loss of tolerance. ^35^	PDCD4, PTEN, SPRY1	**miR-21**
Moderate–strong	miRTarBase	Experimentally validated	Inhibits DNA methylation in T-cells, leading in the overexpression of usually suppressed immune genes and increased autoreactivity. ^36^	DNMT1	**miR-148a**
Moderate	TargetScan	Computationally predicted (TargetScan)	Alters T-cell signaling and migration; dysregulation is associated with higher disease activity. ^37^	ADCY9, RAC1, signaling molecules	**miR-142-3p/5p**
Moderate	TargetScan	Computationally predicted (TargetScan)	Controls DNA methylation and extracellular matrix turnover; links to lupus nephritis and fibrotic tissue damage. ^38^	DNMT3A/B, fibrosis-related genes	**miR-29b/miR-29c**
Moderate	TargetScan	Computationally predicted (TargetScan)	Modulating T-cell differentiation may inhibit regulatory T-cell development, reducing peripheral tolerance. ^39^	FOXP3 regulators	**miR-31**
Moderate	TargetScan	Computationally predicted (TargetScan)	Regulates B-cell maturation and plasma-cell differentiation; abnormal levels disrupt normal antibody production. ^40^	c-Myb, PRDM1	**miR-150**
Moderate	TargetScan	Computationally predicted (TargetScan)	Promotes B-cell survival and activation, as well as the development of persistent autoantibodies. ^41^	BAFF, BCL2 family members	**miR-30a**
Moderate	miRTarBase	Experimentally validated	Downregulating the NF-κB pathway leads to increased inflammatory cytokine release. ^42^	Table 2, TAK1, IKKα	**miR-23b**
Moderate	TargetScan	Computationally predicted (TargetScan)	Regulates inflammatory gene expression and activates the innate immune system. ^43^	TLR4, IL6 regulators	**Let-7 family (e.g., let-7c)**
Moderate	miRTarBase	Experimentally validated	Involved in endothelial activation and leukocyte adhesion, which leads to vascular inflammation in SLE. ^44^	VCAM-1, PI3K signaling components	**miR-126**
Limited	TargetScan	Computationally predicted (TargetScan)	Emerging miRNAs that regulate interferon signaling and lymphocyte activation; data is spars. ^45^	Various IFN-related and B-cell regulatory genes	**miR-3148/miR-569/miR-422a/miR-26a**

Research has shown that miRNAs play a critical role in controlling autoimmune disorders including SLE and B-cell responses. miRNAs have various target genes and pathways. However, the functions and processes of miRNAs in SLE remain unclear. A growing number of miRNAs, including miR-7, miR-10a, miR-15b, miR-21, miR-22, and miR-25, have dysregulated expressions in SLE patients. Reduced expression of certain miRNAs correlates negatively with disease activity or interferon scores in SLE. ^27^

Studies have profiled the expression pattern of miRNA in SLE patients. miRNAs such as miR-146a, miR-146b, and miR-155 have been shown to be significantly upregulated in IL-6 and IL-8, producing cells leading to Th17 differentiation and promoting SLE pathogenesis via IL-17. ^46^

The level of miRNA expression depends on the stage of SLE development. In patients at an early stage of SLE, serum levels of miR-145, miR‐31 and miR-410 are lower than in patients with more advanced SLE progression. Furthermore, patients with SLE in the earlier stage have a notably lower level of these miRNAs in serum when compared to healthy controls, and these miRNAs may serve as biomarkers to better differentiate patients at an early stage of SLE from healthy subjects. ^47^

### 10.1 Evidence grading criteria

The strength of evidence was categorized according to the number of supporting studies, type of evidence (in vitro, vivo, or clinical), and reproducibility across independent cohorts. “Strong” evidence indicates consistent findings across multiple clinical and experimental studies, while “moderate” or “limited” evidence reflects fewer studies or lack of functional validation. ^48^

## 11 Integrated miRNA–gene network analysis reveals pathway convergence in SLE

**Fig. 6:**
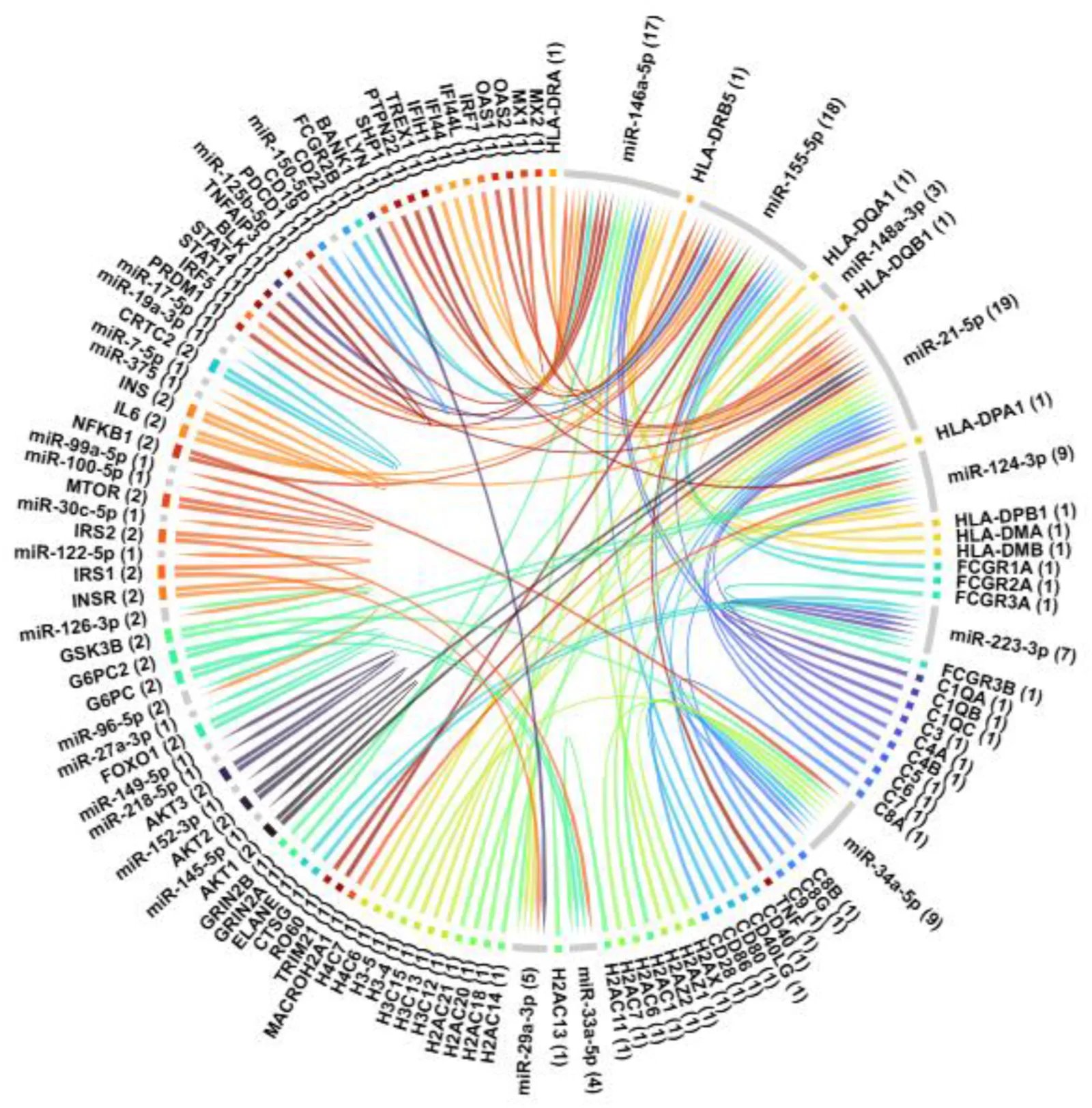
miRNA–target interaction network in SLE.

To further integrate epigenetic regulation with immune network architecture, an miRNA–gene interaction network was constructed to link circulating miRNAs to highly connected hub genes identified through STRING analysis. The network demonstrates that multiple SLE-associated miRNAs converge on a limited set of central immune genes involved in antigen processing and presentation, complement activation, Fc receptor signaling, inflammatory cytokine pathways, and nuclear autoantigen regulation. This integrative view supports a pathway-level model in which miRNAs modulate interconnected immune hubs rather than acting through isolated molecular targets in SLE, ^49^ as shown in Fig. 6 & Table 3.

**Table 3:** Hub genes identified from network analysis and their associated pathways and miRNAs. (STRING database).

Related miRNAs	Key pathway(s)	Main relevance	Node degree	Gene
miR-146a-5p, miR-155-5p	MHC class II antigen processing and presentation	Central hub, antigen presentation	20	HLA-DRA
miR-155-5p, miR-21-5p	MHC class II antigen processing and presentation	Central hub, immune response	18	HLA-DRB5
miR-124-3p, miR-146a-5p	MHC class II pathway	Antigen presentation	15	HLA-DPA1
miR-124-3p, miR-146a-5p	MHC class II pathway	Antigen presentation	15	HLA-DPB1
miR-148a-3p, miR-21-5p	MHC class II antigen processing and presentation	Central hub, immune modulation	17	HLA-DQA1
miR-21-5p, miR-155-5p	MHC class II antigen processing and presentation	Central hub, immune modulation	17	HLA-DQB1
miR-146a-5p	Antigen processing	MHC II loading facilitator	12	HLA-DMA
miR-146a-5p	Antigen processing	MHC II loading facilitator	12	HLA-DMB
miR-146a-5p	MHC class II pathway	Regulator of HLA-DM	10	HLA-DOA
miR-155-5p	MHC class II pathway	Regulator of HLA-DM	10	HLA-DOB

The network illustrates the interactions between dysregulated miRNAs and their target genes involved in SLE pathogenesis. Interactions were obtained from publicly available databases, including miRTarBase (experimentally validated interactions) and TargetScan (computationally predicted targets).

### 11.1 Functional enrichment analysis of SLE-associated hub genes

To further characterize the biological relevance of the identified hub genes, functional enrichment analysis was performed using Gene Ontology (GO) and Kyoto Encyclopedia of Genes and Genomes (KEGG) databases. The analysis revealed significant enrichment of pathways associated with immune regulation and autoimmune disease pathogenesis.

The most enriched biological processes included antigen processing and presentation, regulation of adaptive immune responses, cytokine-mediated signaling pathways, interferon signaling, and leukocyte activation. KEGG pathway analysis highlighted several immune-related pathways, including T-cell receptor signaling, B-cell receptor signaling, Toll-like receptor signaling, NF-κB signaling, cytokine–cytokine receptor interaction, and pathways associated with SLE.

These findings support the network-based observations presented in this review and further emphasize the central role of dysregulated immune signaling pathways in SLE pathogenesis. A summary of the enriched pathways is provided in Supplementary Table S1.

## 12 Clinical utility and limitations of miRNA biomarkers

While circulating miRNAs have shown promise as potential biomarkers for SLE diagnosis and disease monitoring, several challenges limit their clinical application. Compared with conventional biomarkers such as anti-dsDNA antibodies and complement levels (C3, C4), miRNAs may offer higher sensitivity in detecting early disease changes; however, their specificity remains variable across studies. ^50^

Additionally, variability in sample processing, normalization methods, and detection platforms can affect reproducibility. Inter-individual variability and differences in disease activity further complicate their clinical interpretation. Standardization of assay protocols and large-scale validation studies are required before miRNA-based biomarkers can be reliably implemented in clinical practice. ^51^

## 13 miRNA-146a-5p

miR-146a-5p could be a diagnostic biomarker in SLE and may play a functional role in the disease's ocular symptoms. miR-146a-5p is well-known for regulating immune response and inflammation. miR-146a is produced by the activation of toll-like receptor 4 in the NF-kB-dependent signaling pathway, leading to the downregulation of IL-1 receptor-associated kinase 1 and TNF-receptor-associated factor 6. ^52^

miR-146a-5p plays an important role in immunological abnormalities and organ injury of SLE by controlling the disease's inflammation and consequences. miR-146a-5p may serve as a diagnostic marker to distinguish between patients with SLE and have modest diagnostic value. Furthermore, miR-146a-5p is linked to disease symptoms and higher disease activity levels. This highlights the importance of miR-146a-5p and pro-inflammatory cytokines in the immunopathology of SLE. ^28^

## 14 miRNA-155-3p

miR-155-3p is expressed by both immune and infected cells during viral infections. miR-155-3p has a significant impact on antiviral innate and adaptive immune responses. Irregular miR-155-3p expression promotes viral persistence and pathological responses. miR-155 expression is rapidly induced during an infection because pathogen-associated molecular patterns (PAMPs) can operate as inducers of miR-155-3p expression, and its expression is also stimulated by TLR agonists. ^53^

As a biomarker for the progression and activity of the disease, miR-155 overexpression is seen in SLE. As the condition progresses, it also poses a risk for kidney involvement. Research has shown that miRNAs transported by exosomes may control the inflammatory reaction to endotoxins, and endogenous miRNAs were a mechanism for controlling the inflammatory response through functional cell-to-cell transfer. ^54^

In SLE, miRNA-155 plays a significant role. The condition is still incurable, even though there have been advances in treating SLE. As a result, the use of epigenetics in the search for new medicines has recently gained traction. Some messenger RNAs may have competing roles, and a single miRNA may target many mRNAs. One biomarker in SLE is miR-155. ^55^

## 15 miRNA-192-5p

Recent research has shown that miR-192-5p, a member of the miR-192 family, is involved in multiple human diseases, particularly malignancies of the lung, liver, and breast. Importantly, miR-192-5p levels are prevalent in biofluids such as serum and urine, and exosomal levels of miR-192-5p in circulation can help with the diagnosis and prognosis of a variety of disorders, including chronic hepatitis B (CHB) infection. As a result, miR-192-5p is an essential miRNA for the evolution of human diseases, as it can be regulated by other important regulatory molecules, influencing the expression of downstream genes. ^56^

In addition to genetics and environment, epigenetic factors, particularly miRNAs, have a role in disease development. Furthermore, plasma levels of miR-192 were considerably higher in SLE patients than in controls. There was no significant correlation between circulating miR-192 and clinical parameters. miR-192 may contribute to the pathophysiology of SLE. ^8^

## 16 miRNA-21-5p

miRNA-21 is one of the most prevalent and conserved miRNAs known. It is found in virtually all cells and plays critical regulatory roles in health and illness. In miRNA research based on physiological fluids, it is widely claimed to be a biomarker of diseases such as cancer and heart disease miR-21 expression varies across 78 cell types. miR-21 is highly expressed in numerous cell types, however it is most abundant in macrophages, monocytes, and dendritic cells. ^57^

The stability of miRNAs in the bloodstream is well established, and serum and plasma levels are connected to disease progression. As a result, they may be biomarker candidates. The role of miRNAs in the formation of proliferative lymph nodes has been widely researched. Recent research focused on the correlation between the miRNAs and SLE risk has been described in recent years, particularly the interaction between miR-21 and SLE.^19^

## 17 Therapeutic targeting strategies and clinical translation of miRNAs in SLE

Despite the promising role of miRNAs as therapeutic targets in SLE, several challenges limit their clinical translation. One of the major obstacles is the efficient and targeted delivery of miRNA-based therapeutics. miRNAs are inherently unstable in circulation and are susceptible to rapid degradation by nucleases, necessitating the use of protective delivery systems. ^58^

Various delivery platforms have been developed, including miRNA mimics to restore downregulated miRNAs and antagomirs or locked nucleic acid (LNA)-based inhibitors to suppress overexpressed miRNAs. In addition, nanoparticle-based delivery systems and viral vectors have shown potential in enhancing stability, cellular uptake, and tissue-specific targeting. However, issues such as off-target effects, toxicity, and immunogenic responses remain significant concerns. ^59^

From a regulatory perspective, miRNA-based therapeutics face challenges similar to other nucleic acid-based drugs, including stringent requirements for preclinical safety evaluation, pharmacokinetics, and long-term toxicity. The transition from preclinical models to human clinical trials is further complicated by differences in miRNA expression patterns between species. ^60^

Moreover, the development timeline for miRNA therapeutics is relatively long, requiring extensive validation through phased clinical trials to ensure safety and efficacy. Standardization of manufacturing processes and scalability also represent critical barriers to clinical implementation. ^61^ Collectively, while miRNA-based strategies hold great promise for precision medicine in SLE, overcoming these translational challenges is essential before their routine clinical application can be realized. ^62^

### 17.1 Comparison with existing therapeutic approaches and implementation barriers

Current treatment strategies for SLE primarily rely on immunosuppressive agents, corticosteroids, and biologics targeting specific immune pathways. While these approaches can control disease activity, they are often associated with significant side effects and variable patient responses. ^63^

In contrast, miRNA-based therapies offer a more targeted approach by modulating gene expression at the post-transcriptional level and potentially affecting multiple disease pathways simultaneously. However, despite this advantage, several barriers hinder their clinical implementation, including challenges in targeted delivery, risk of off-target effects, high development costs, and limited long-term safety data. ^64^

Moreover, translating miRNA-based therapeutics into routine clinical use requires overcoming regulatory challenges and ensuring scalability and cost-effectiveness. ^65^

### 17.2 miRNA-based therapeutic strategies

miRNA-based therapeutics aim to restore or inhibit the function of dysregulated miRNAs in SLE. miRNA mimics are designed to replenish downregulated miRNAs, whereas antagomirs and locked nucleic acid (LNA)-based inhibitors are used to suppress overexpressed miRNAs. These approaches have shown promising results in modulating immune responses in preclinical models. ^66^

### 17.3 Delivery systems for miRNA therapeutics

Efficient delivery remains a major challenge for miRNA-based therapies. Various delivery systems, including lipid nanoparticles, polymer-based carriers, and viral vectors, have been explored to improve miRNA stability and targeted delivery. Nanoparticle-based systems, in particular, offer advantages in protecting miRNAs from degradation and improving cellular uptake. ^67^

### 17.4 Clinical translation and therapeutic challenges

Despite the promising role of miRNAs as therapeutic targets in SLE, several challenges limit their clinical translation. One of the major obstacles is the efficient and targeted delivery of miRNA-based therapeutics, as miRNAs are inherently unstable and susceptible to degradation. ^58^

Various delivery platforms have been explored, including miRNA mimics, antagomirs, and nanoparticle-based systems; however, off-target effects, toxicity, and immunogenicity remain significant concerns. From a regulatory perspective, miRNA-based therapeutics require extensive preclinical validation, pharmacokinetic profiling, and long-term safety assessment.

Moreover, the transition from preclinical models to clinical trials is complicated by biological variability, and the overall development timeline is relatively long. Standardization and large-scale manufacturing also represent key barriers to clinical implementation. ^68^

### 17.5 Future perspectives of miRNA therapeutics in SLE

Future research should focus on improving delivery efficiency, minimizing off-target effects, and enhancing the safety profile of miRNA-based therapies. Advances in nanotechnology and precision medicine may accelerate the development of targeted therapeutic strategies. Additionally, well-designed clinical trials are required to validate the efficacy and safety of miRNA-based interventions in SLE patients. Integrating miRNA profiling into personalized medicine approaches may offer new opportunities for disease management and therapeutic optimization. ^69^

## 18 Conclusion

The identification of dysregulated miRNAs in SLE has advanced our understanding of disease mechanisms at the molecular level. Their ability to regulate key immune pathways supports their potential utility as biomarkers for disease diagnosis and monitoring, as well as prospective therapeutic targets. The network-based approach adopted in this review provides an integrative framework illustrating how circulating miRNAs converge on central immune pathways implicated in SLE, emphasizing coordinated pathway dysregulation rather than isolated molecular effects. While miRNA-based strategies hold promises for precision medicine in SLE, large-scale validation studies and clinical trials are required. Future research should further explore interactions between miRNAs and other epigenetic mechanisms to support clinical translation and improved patient management.

## Availability of data and materials

All data generated or analyzed during this study are included in this review article.

## Funding

No funds were received for this project, and the study was self-funded.

## CRediT authorship contribution statement

**Mohamed M. Sadaty:** Writing – original draft, Formal analysis. **Rana Mohamed:** Writing – original draft, Investigation. **Emad El-Zayat:** Writing – review & editing, Supervision. **Salma M. Mekhemer:** Writing – review & editing, Project administration. **Amira El-Ansary:** Writing – review & editing, Supervision. **Nahla O. Mousa:** Writing – original draft, Conceptualization.

## Declaration of competing interest

The authors declare that they have no competing interests.
