# Supplementary material for: MicroRNAs in systemic lupus erythematosus: Molecular networks, pathway dysregulation, and therapeutic targeting strategies
Source: Biotechnol Notes. 2026 Sep 1;7:183–94. doi: 10.1016/j.biotno.2026.07.002 (PMC13562414; doi:10.1016/j.biotno.2026.07.002)
Supplement: Supplementary file 1 [file mmc1.docx]

**Supplemental Files**

**Supplementary Table S1.**

**Representative GO and KEGG enrichment results of SLE-associated hub genes.**

| **Enriched pathway / biological process** | **Gene count** | **Representative genes** |
| --- | --- | --- |
| Antigen processing and presentation | 10 | HLA-DRA, HLA-DRB5, HLA-DPA1, HLA-DPB1, HLA-DQA1 |
| T-cell receptor signaling pathway | 7 | CD40LG, HLA-DRA, HLA-DQA1, HLA-DQB1 |
| B-cell receptor signaling pathway | 6 | CD40LG, FCGR2A, FCGR3A, HLA-DRA |
| Toll-like receptor signaling pathway | 5 | IRAK1, TRAF6, TLR4, IFN-related genes |
| NF-κB signaling pathway | 5 | TRAF6, IRAK1, TAB2, TAK1, IKKα |
| Cytokine–cytokine receptor interaction | 8 | IL6, IL8, IFN-regulated genes |
| Type I interferon signaling pathway | 6 | IFN-regulated genes, IRF-related genes |
| Regulation of adaptive immune response | 9 | HLA class II genes, CD40LG |
| Leukocyte activation | 8 | FCGR2A, FCGR3A, HLA genes |
| Systemic lupus erythematosus pathway | 11 | HLA-DRA, HLA-DRB5, HLA-DPA1, HLA-DPB1, HLA-DQA1, HLA-DQB1 |
